# The Effects of Resveratrol on Telomeres and Post Myocardial Infarction Remodeling

**DOI:** 10.7759/cureus.11482

**Published:** 2020-11-14

**Authors:** Sai Dheeraj Gutlapalli, Varshitha Kondapaneni, Ijeoma A Toulassi, Sujan Poudel, Mehwish Zeb, Jinal Choudhari, Ivan Cancarevic

**Affiliations:** 1 Internal Medicine, California Institute of Behavioral Neurosciences & Psychology, Fairfield, USA; 2 Family Medicine, California Institute of Behavioral Neurosciences & Psychology, Fairfield, USA; 3 Psychiatry and Behavioral Sciences, California Institute of Behavioral Neurosciences & Psychology, Fairfield, USA; 4 Pediatrics, Khyber Teaching Hospital, Peshawar, PAK

**Keywords:** resveratrol, telomeres, post-myocardial infarction

## Abstract

Post myocardial infarction (MI) remodeling is the term used to define the changes in cardiac musculature after sustaining an ischemic injury. These changes decrease myocardial function and ultimately lead to heart failure. We review the contributing factors to post-MI remodeling, its association with telomere biology, as well as a myriad of other factors affecting aging and telomere length in relation to cardiovascular health. The main focus is on the effects of resveratrol in the cardiovascular system and its potential for therapeutic use in preventing long-term cardiovascular morbidity and mortality. We tried to answer important questions regarding the potential for resveratrol as a therapeutic drug to prevent adverse post-MI remodeling. In our search, we gathered 62 studies and narrowed our data down to 44 studies. The database used was PubMed, and the keywords used are "Resveratrol", "Telomere", "Post Myocardial Infarction". All the studies were carefully screened for relevant articles regarding our topic manually, Articles related to a positive association between resveratrol and its anti-aging, cardioprotective effects have been included in our study, as we could not find any articles in our search which showed a negative correlation. Our review concluded that resveratrol had pro-telomerase effects which could counter the development of adverse post-MI remodeling. Therefore resveratrol could be a useful therapeutic add-on drug to prevent cardiovascular disease. It is essential that further research including observational and large-scale clinical trials should be conducted to increase our understanding of the efficacy and viability of these novel therapeutic interventions.

## Introduction and background

Myocardial Infarction (MI) is one of the most common causes of cardiovascular morbidity and mortality in the world [1]. Approximately 45,000 myocardial infarctions occur every day across the globe [1]. We know that in many cases, after the ischemic injury to the myocardium, adverse post-MI remodeling occurs, leading to poor long-term outcomes [2]. Therefore for effective prevention of long-term sequelae, a more detailed understanding of the aging-related factors like telomere shortening, oxidative damage, autophagy, and cellular senescence is required [2].

Resveratrol is a phenolic phytochemical derived from grapes [3]. It is a bioactive substance that is physiologically neuroprotective, cardioprotective, nephroprotective, anti-neoplastic, along with a broad spectrum of antioxidant, anti-aging effects [3]. It is well known that resveratrol has anti-inflammatory and vascular protective effects in different experimental models [4].

We know that the main biological determinants of aging are the heterochromatic repeat regions at the ends of the eukaryotic chromosomes called telomeres [5]. Telomere shortening is an important phenomenon in aging, along with DNA damage, oxidative stress, inflammation, and mitogenic signals [6]. Critically short telomeres trigger cell death, and the rate of telomere shortening is accelerated in cells subjected to oxidative stress and damage, like in scenarios of ischemic injury due to MI [5]. The enzyme responsible for telomere elongation and repair is telomerase; therefore enhancing the telomerase activity is an important factor in preventing the cellular aging process [7]. Resveratrol has been shown to have attenuating effects on the progression of the aging process in cardiomyocytes by suppressing mitochondrial elongation and activation of Parkin and PINK1 Proteins [4]. Studies have shown that resveratrol significantly increases the telomerase activity by increasing the expression of the catalytic subunit of human telomerase reverse transcriptase (hTRET) in a dose-dependent manner [8].

The main discussion of this review is regarding the protective effects of resveratrol on telomere shortening in post-myocardial infarction patients. We try to understand if resveratrol can be effective in slowing or preventing cardiac damage and improve long term prognosis post-MI. This article mainly discusses the various factors contributing to aging and oxidative damage in the cardiovascular system and the possible preventive options; the focus is going to be on the viability of resveratrol as a supplement for the prevention of oxidative damage in the heart post-MI and preventing cardiomyocyte aging and senescence. The relevant data for our literature review was gathered from PubMed database, using three keywords “Resveratrol“ ,“Telomere", "Post-Myocardial Infarction", the search was performed by combined keyword search using MeSH strategy. We manually screened and included all the relevant articles we could find since inception till September 20th, 2020.

## Review

Significance of telomeres in aging 

**Figure 1:**
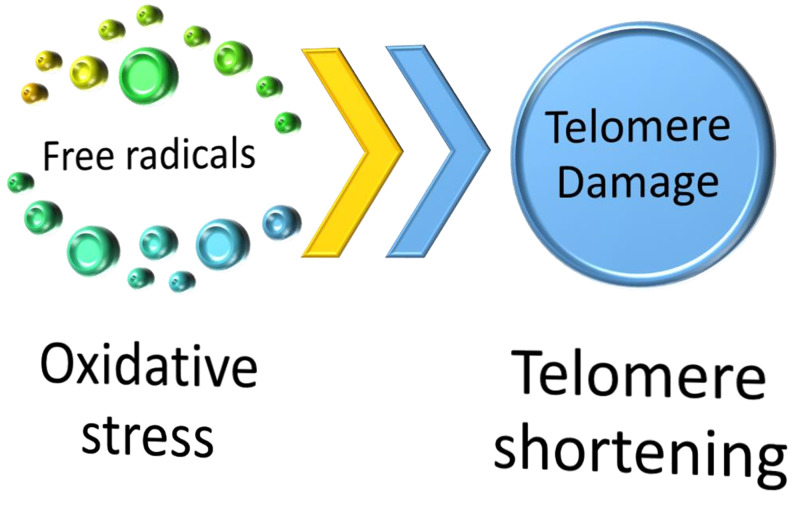
Effect of oxidative stress on telomere

In recent years telomere biology has emerged as an essential player in the aging and disease process [9]. Telomeres are biomarkers for aging; they are specialized nucleoprotein structures at the ends of chromosomes, the shortening of telomeres is a natural phenomenon that signifies aging of the cell, but in conditions of DNA damage, oxidative stress, ischemic injury, or any other pathological process the rate of telomere shortening is accelerated causing rapid cellular aging and death [6,7]. Telomerase is an enzyme in the human body that promotes the elongation and maintenance of telomere length [6,7]. Therefore substances that have pro telomerase activity are beneficial in maintaining the health of telomeres, thus preventing cell senescence and cellular aging [6,7]. Kordinas et al. report that telomere/telomerase dysfunction is being studied in relation to the development of chronic conditions such as cardiovascular disease in many laboratories worldwide [7]. Cellular senescence is the arrest of the cell in the cell cycle (G₀) phase [6]. Senescence is driven by many factors oxidative stress, mitogenic signals, DNA damage and repair mechanisms, inflammation, and importantly, telomere shortening; telomeres are shortened by each cell division until a critical length when the cellular dysfunction occurs; Telomere length is considered a marker for cardiovascular aging [6]. Zhang et al. reported that apart from shortened and dysfunctional telomeres, cells undergoing senescence are also associated with increased activity of transcription factor NF-κB and increased expression of inflammatory cytokines such as TNF-α, IL-6, and IFN-γ in circulating macrophages [5]. Importantly telomerase is a reverse transcriptase involved in modulating NF-κB activity [5]. Cardiovascular disease such as atherosclerosis, hypertension, heart failure is associated with short leukocyte telomeres [6]. According to Longo et al. there was a consensus that there is sufficient evidence that aging interventions will delay and prevent disease onset for many chronic conditions of adult and old age like cardiovascular diseases [10]. According to Blagosklonny, aging is neither driven by the accumulation of molecular damage or random damage of any kind [11]. They studied predictions of a new theory of quasi-programmed hyperfunction, have already been confirmed, and by clinically-available drugs slowing aging and delaying diseases in animals [11]. Bhattacharyya et al. studied the usefulness of shortening telomeres as a potential biomarker of CAD [12]. Clinical research evidence highlighting the role of telomere shortening in CAD is well known in different ethnic populations of the world [12]. Establishing a well-standardized and accurate method of evaluating telomere length is essential before its routine use in preventive cardiology [12]. According to Yegorov et al. accelerated shortening of telomeres in all cell types is seen in people exposed to chronic stress, and they age rapidly; inflammation is a major feature of stress, along with aging, together causing the phenomenon of inflammaging [13]. Oxidative stress is due to the over-production of reactive oxygen species (ROS) that can damage various tissues [13]. Barbosa et al. studied the importance of autophagy; it is an essential protein turnover pathway; it delivers cellular components for lysosomal degradation or recycling [14]. This process maintains the cellular homeostasis, its dysregulation leads to physiological/pathological alterations [14]. Autophagic activity decreases with aging, leading to the accumulation of damaged macromolecules, intracellular organelles [14]. Failure of autophagic pathways worsens aging-related diseases like cardiovascular, cerebrovascular disease, or cancer [14]. It has been hypothesized in different organisms that proper maintenance of autophagic activity leads to extended longevity [14]. The effects of free radicals and oxidative stress on telomeres are shown in Figure 1.

Based on the above discussions, It is understood that telomere length is an important marker for aging, it is also clear that increasing human telomerase activity is essential to preventing aging and oxidative damage in many of the organ systems, including the cardiovascular system; therefore substances which have pro telomerase effects could be useful in countering the adverse cardiac remodeling post-MI.

Factors influencing myocardial aging and remodeling

Cardiac aging causes compromised cardiac function; altered cardiac morphology leads to tissue degeneration and heart failure [15]. A polymorphic mitochondrial enzyme Aldehyde Dehydrogenase 2 (ALDH2) governs cardiac function [15]. Studies by Zhang et al. focused on the role of ALDH2 in cardiac aging [15]. It was noted that myocardial function and intracellular Ca(2+) function declined with aging; these effects were accentuated by ALDH2 [15]. Cardiac hypertrophy, interstitial fibrosis, and greater left ventricular wall thickness were seen in aged mice in these studies [15]. Aging induced O2(-) generation and cardiomyocyte dysfunction were accentuated by treatment with ALDH2 activator Alda-1, and these effects were decreased by cotreatment with resveratrol or activators of AICAR, Sirtuin-1 (SIRT1), AMPK, SRT1720 [15]. Their data showed ALDH2 enzyme increased adverse cardiac remodeling and dysfunction with aging by the AMPK/SIRT1-mediated mitochondrial damage [15]. Cianflone et al. reported that adult cardiac stem cells (CSC) senescence is associated with a decline in myocardial tissue repair [16]. CSC Senescence is represented by its replicative limit, which is mainly determined by telomere shortening, proper regulation of p53/p16INK4/Rb molecular pathways, chromatin remodeling [16]. They concluded that senescent cells significantly influence the outcome of cardiac diseases, therefore novel therapies targeting clearance of senescent cells and reversing the senescent phenotype should be explored [16]. According to Pulakat et al. aging is accelerated by obesity and diabetes and induces cardiovascular changes characterized by pathological remodeling associated with myocardial and vascular fibrosis, stiffness and hypertrophy, macro and microcirculatory impairment, left ventricular diastolic dysfunction leading to heart failure, and ultimately cardiovascular cell death [17]. They noted that many longevity treatments are related to the induction of autophagy, activation of the Sirtuin pathway, AMPK Activation, inhibition of mTOR (mechanistic target of rapamycin) [17]. Such treatments focus on calorie restriction and drugs like resveratrol, metformin, rapamycin, aspirin [17]. Autophagy is highly regulated in cardiac tissue; excessive autophagy may cause cardiomyopathy and heart failure; they concluded that regulation of autophagy is important for preventing myocardial aging [17]. According to Shimizu et al. senescent cells are pro-inflammatory and cause chronic sterile inflammation [18]. Senescent cell accumulation is considered pathogenic in the cardiovascular system as it leads to vascular remodeling; novel approaches have allowed for eliminating senescent cells in vivo and in vitro; senolysis is the term used for this process [18]. Senolysis could reverse aging-related cardiovascular pathologies without potential for tumorigenesis [18]. Cardiac myocytes ( have the most abundant mitochondrial population ) are terminally differentiated cells with low regenerative capacity in the adult [19]. Cardiomyocyte mitochondrial dysfunction is a main feature of the aging heart [19]. Lin et al. studied aging and cardiac pathologies in association with increased senescence in the heart [19]. The unique cellular composition of the heart, especially the functional properties of cardiomyocytes, need to be considered when designing therapeutics to target cardiac aging [19]. They reviewed recent findings regarding key factors regulating cell senescence, mitochondrial health as well as cardiomyocyte rejuvenation [19]. According to Lapuente et al. inadequate fruit and vegetable is a risk factor for cardiovascular disease [20]. They concluded that well-designed, large-scale, long-term studies are needed to better understand the role fruit/vegetable consumption in atherosclerosis [20]. Aging is an important predisposing factor for fibrotic heart disease [21]. Murtha et al. reported that processes of senescence, inflammaging, autophagy, and mitochondrial dysfunction diminish the cardiac regenerative capacity and play a major role in cardiac fibrosis [21]. Studies by Cianflone et al. showed that aging is the single most important risk factor for the development of heart disease [22]. Pathological molecular changes in myocardial tissue homeostasis results in deterioration in cardiac structure and function [22]. The role of endogenous cardiac stem cells (CSC) activation is a recent discovery in the biology of mammalian myocardium, which proves that the adult heart is a dynamic organ where cardiac cells continuously die and are replaced by CSC progeny [22]. Senescence in CSCs is characterized by oxidative damage, an increase in ROS production, loss of telomere/telomerase function [22]. Regardless, the aged myocardium retains endogenously functional CSC cohort, which is resistant to senescence occurring with age [22]. It seems the latter CSC aging is a reversible autonomous cellular process [22]. CSC aging may also be a programmed cell cycle-dependent phenomenon affecting all endogenous CSCs; meaning that loss of regenerative capacity seen in CSCs is an inevitable process [22]. Therefore, better understanding of both biological viewpoints is needed to develop CSC-based interventions to prevent cardiovascular aging [22]. Miyamoto et al. reported that cardiac autophagy decreases with aging, leading to accumulation of dysfunctional mitochondria and misfolded proteins in the heart [23]. Therefore inhibition of autophagy accelerates cardiac aging, and induction of autophagy causes improvement in cardiac function [23]. Autophagy is a potential target for therapies of age-related cardiac dysfunction [23]. According to Ungvari et al. the pathophysiological roles of cellular and molecular mechanisms of aging such as oxidative stress-related damage, mitochondrial dysfunction, impaired resistance to molecular stressors, chronic low-grade inflammation, genomic instability, cellular senescence, epigenetic alterations, loss of protein homeostasis, dysregulated nutrient sensing, and stem cell dysfunction in the vascular system are contributors to cardiovascular disease [24]. They studied the significance of progeronic and antigeronic circulating factors in relation to the development of vascular aging phenotypes [24]. Another study by Ungvari et al. showed that age-related endothelial dysfunction associated with abnormal angiogenesis and the resultant pathological remodeling in microvasculature contributed to decreased tissue perfusion and accelerated functional decline in elderly [25]. Therefore a functional endothelium is critical for tissue perfusion and long-term cardiovascular health [25]. Kaur et al. reviewed studies which utilized autologous adult stem cells as a treatment for healing cardiac tissue post-MI; they focused on three strategies that are effective in regenerating aged cardiac stem cells, (1) genetic modification, (2) pharmaceuticals, (3) extracellular factor optimization [26].

**Figure 2:**
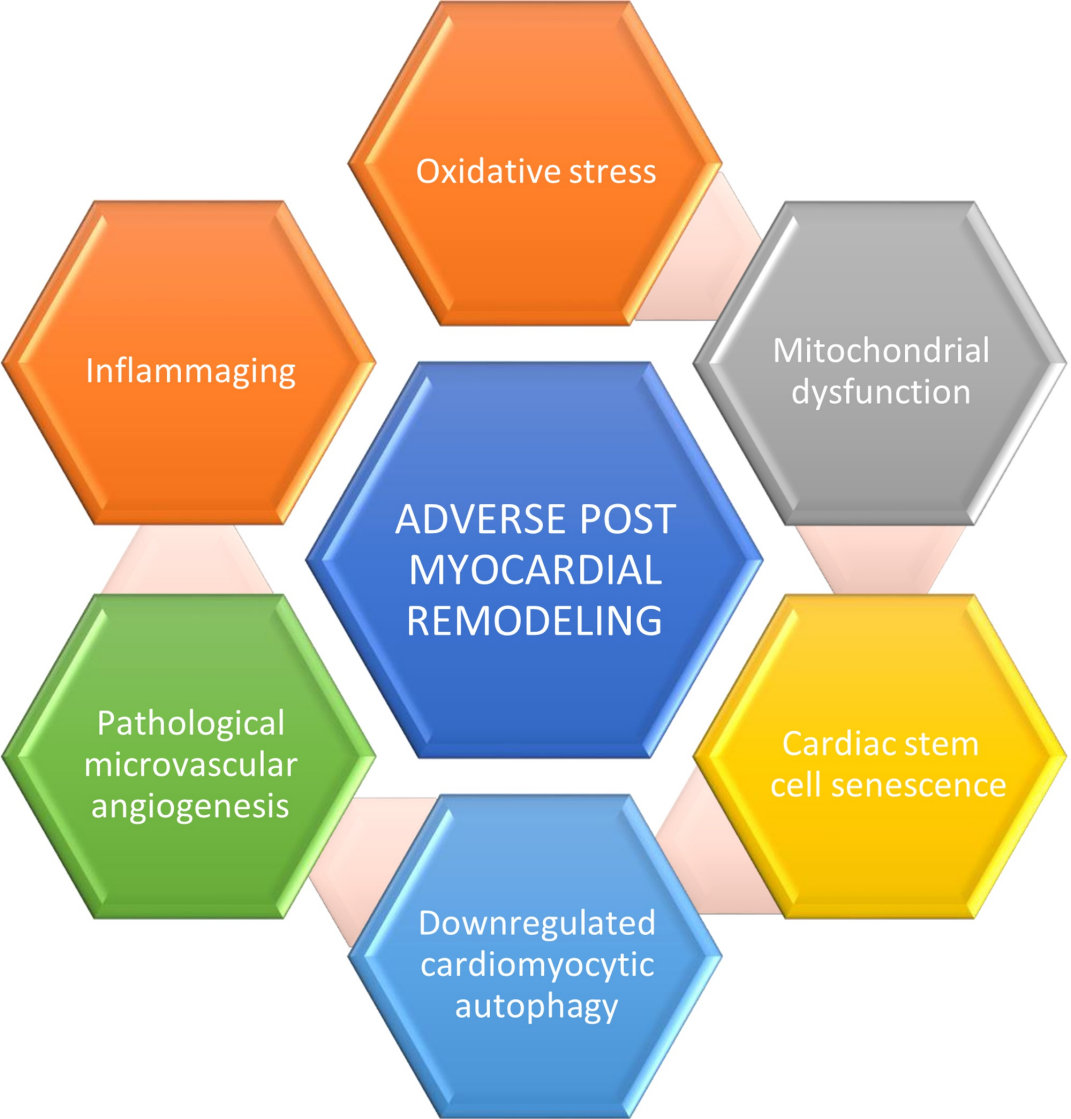
Factors contributing to adverse post-myocardial remodeling

Developmental programming and aging are associated with endothelial dysfunction and oxidative damage [27]. Allison et al. studied rodent models based on programmed cardiovascular dysfunction; they determined the vascular telomere length and endothelial function in young (four-month-old ) and aged adult (15-month-old) offspring of normoxic and hypoxic pregnancy with and without maternal antioxidant supplementation [27]. Their data showed evidence of divergence of mechanistic pathways involved in cardiovascular aging and the decelerating effect of antioxidants prior to birth on the developmental programming of cardiovascular diseases [27]. Chiao et al. studied cardiac aging-associated phenotypic changes and the underlying molecular mechanism; their focus was on the recent advances in therapeutic interventions in delaying cardiac aging [28]. Wang et al. stated that proinflammatory remodeling in the heart, and large vessels are driven by aging-related stress and angiotensin II [29]. Changes like vascular wall stiffening, suboptimal ventricular arterial coupling, systolic hypertension are seen in the aging cardiovasculature and termed the silent syndrome [29]. These changes are due to proinflammatory cardiovascular cells, and local inflammatory signals coupled between arteries and the heart due to common messenger pathways in a closed circulatory system; it was concluded that novel treatments targeting pro-inflammatory signaling pathways are promising therapies for heart failure [29]. Rohrbach et al. reported from observations in multiple species, including yeasts to rodents, that the most reliable intervention to increase lifespan and prevent aging-associated disorders is calorie restriction (CR) [30]. Both short and long-term CR has cardioprotective effects against ischemic injury in young and old rodents [30]. Human trials have shown that CR improves cardiovascular function and causes regression of cardiac senescence [30]. Known mediators of CR are AMP-activated PK, sirtuins, adiponectin, and NO [30]. Mitochondria also plays an important role in CR-induced protective effects against ischemic/reperfusion injury [30]. The CR-mimetic drug resveratrol has cardiovascular protective effects [30]. Aging in the human heart includes hypertrophy of the left ventricle, valvular degeneration, cardiac fibrosis, diastolic dysfunction, atrial fibrillation, decreased exercise tolerance [31]. Dai et al. studied the role of mitochondrial oxidative stress in the pathology of CVDs and the mechanisms causing heart failure in elderly, a better understanding of molecular mechanism of cardiac aging is needed to formulate novel anti-aging strategies for the promotion of healthy cardiac aging [31]. Fontana et al. reviewed associations between cardiac function, heart disease, and growth factors, focusing on the cardioprotective effects of growth factors in animal and human models [32]. Bachschmid et al. studied “the free radical theory of aging” and concluded that oxygen and nitrogen free radicals are important in regulating vascular homeostasis as essential signaling molecules [33]. Stress and lower socioeconomic status causes increased morbidity and mortality [34]. According to Razzoli et al. cellular senescence is a potential mechanism linking long-term stress to aging-related morbidity [34]. The causal relationship of long-term social stress on shorter lifespan, higher cardiovascular disease risk has been established in mice [34]. Idikio et al. studied the effect of normal aging and events of post-MI remodeling, including the cardio-mechanical and neuro-hormonal factors like immunosenescence, regeneration of stem-cells, telomere shortening, oxidative stress, antiaging hormones like melatonin and klotho, diet, sirtuins and concluded that advances in stem cell repair of myocardial infarcts need further research [2]. The factors associated with adverse post-MI remodeling are shown in Figure 2.

According to the data gathered from the studies we reviewed, the major factors influencing post-MI remodeling are oxidative stress, mitochondrial dysfunction, and CSC senescence/dysfunction. The high concentration of mitochondria in cardiomyocytes makes them more prone to oxidative damage due to mitochondrial dysfunction. Other factors promoting adverse myocardial remodeling are inflammaging, pathological angiogenesis, downregulation of cardiomyocyte autophagy. Important associated factors are telomere shortening, immunosenescence, angiotensin II, psychological stress, sirtuins, and abnormal microvascular remodeling. Therapeutic advances which have shown promise in preventing adverse myocardial remodeling, especially post MI remodeling include compounds like resveratrol, senolysis therapy, calorie restriction, anti-aging hormones like melatonin, autologous stem cell therapy, high fruit diet, and other antioxidants.

Resveratrol and its effects

Polyphenols like resveratrol, curcumin, epigallocatechin gallate (EGCG) have protective effects on cardiovascular health [35]. Studies revealed that resveratrol increased endothelial progenitor cell (EPC) number and function [8]. Wang et al. investigated the effects of resveratrol on EPC senescence and found that resveratrol inhibited EPC senescence in a dose-dependent manner [8]. Telomerase activity was also significantly increased in a dose-dependent manner through catalytic subunit hTRET induction caused by resveratrol [8]. Tousian et al. reviewed 52 articles in their study regarding cellular senescence and protective effects of natural substances and found resveratrol and ginseng are the safest and most studied agents in phytotherapy for preventing stem cell senescence and cardiovascular aging [36]. The main molecular target of these agents were antioxidant enzymes and also telomerase to preserve genomic stability [36]. They concluding that resveratrol and ginseng are good choices for long term clinical use as anti-aging supplements [36]. Malhotra et al. studied the antioxidant, cytoprotective, and anti-inflammatory properties of resveratrol on atherosclerosis, cardiac hypertrophy, and ischemic reperfusion injury associated with MI [3]. They also reviewed the pharmacokinetics of commercial resveratrol formulations [3]. According to Komici et al. phytochemicals like resveratrol are an innovative strategy to decrease cardiovascular risk factors, clinical evidence supports the cardioprotective effects of resveratrol [37]. They noted that further research should be conducted on the effects of resveratrol on cardiac remodeling [37]. They also studied the effects of resveratrol in HF therapy and the difference in sex-gender-oriented responses to treatment [37]. A review by Lapuente et al. concluded that bioactive substances in fruits and vegetables like resveratrol have a protective effect on the development of atherosclerosis [20]. Mediterranean diet (MeDi) style was studied by Tuttolomondo et al. MeDi was associated with low levels of inflammatory markers, it had protective role in the cardiac and cerebral vasculature [38]. Positive effects were observed in cardiovascular risk factors like waist circumference, body mass index, lipid metabolism, blood pressure, inflammation, diabetes in several randomized control trials (RCTs) comparing both MeDi and low-fat diet [38]. Several other studies showed a decrease in the incidence of heart failure in adherence to MeDi; important studies such as PREDIMED showed a lower incidence of MI among patients on MeDi supplemented with nuts and virgin olive oil as compared to those on a low-fat diet [38]. Senoner et al. reported that a stable balance in levels of ROS and antioxidants is needed for the normal function of any cell, a minimum concentration of ROS is essential for certain cell functions, and excess in ROS leads to damage to DNA, proteins, lipids in the cell causing cell death [39]. Increased ROS levels cause a lowering in nitric oxide levels leading to vasoconstriction, and a rise in blood pressure; myocardial calcium homeostasis is negatively altered by ROS, leading to arrhythmias, ROS also promotes adverse cardiac remodeling through hypertrophic signaling, accelerated atherosclerosis [39]. An antioxidant-rich diet is therefore beneficial for preventing the effects of ROS in the cardiovascular system [39]. Studies by Aguilar-Alonso et al. on oxidative stress markers (total lipoperoxidation and nitric oxide (NO) levels) as well as catalase activity and superoxide dismutase (SOD) in aging rat cardiomyocytes treated with resveratrol showed a decrease in NO Levels and total lipoperoxidation, but the activity of catalase and SOD was not significantly affected, and it was concluded that the anti-aging and cardioprotective properties of resveratrol are mediated mainly by its antioxidant effects [40]. 

**Figure 3:**
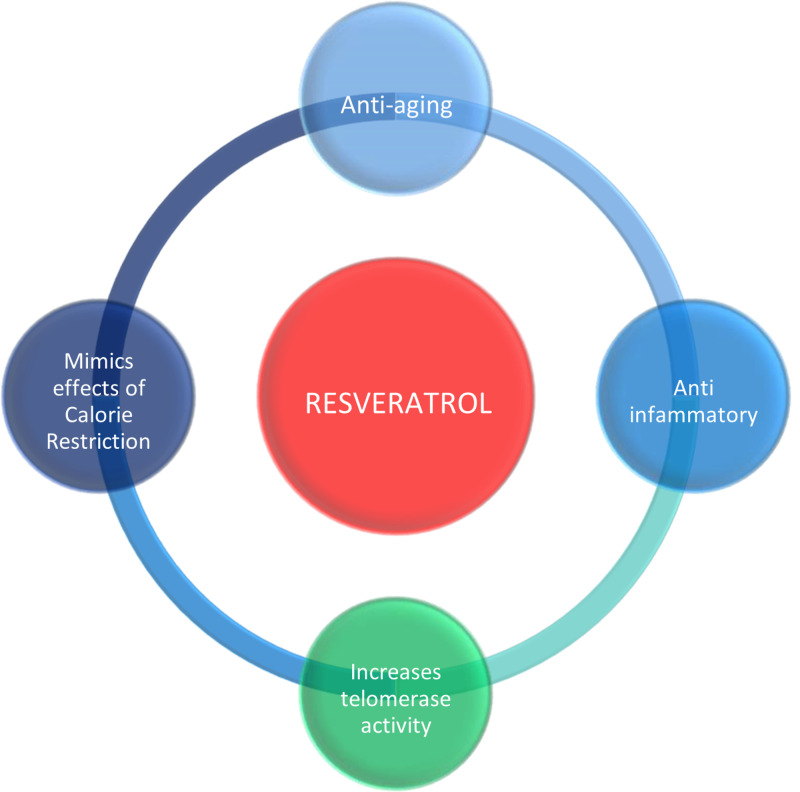
Effects of resveratrol

Studies by Ren et al. showed that resveratrol exerts cardioprotective effects and has anti-aging properties in multiple experimental models [4]. They studied the effects of resveratrol on mitochondrial morphology and depolarisation, and on expressions of LC3, Parkin, PINK1, Drp1 in H9c2 cells after treatment with D-galactose to induce a state of senescence in cardiomyocytes [4]. Their findings clearly showed the effect of resveratrol in slowing cardiovascular aging [4]. Zhang E et al. investigated the SIRT1 Axis Involvement in the protective effects of metformin, resveratrol, other potent SIRT1 Activators in relation to “Metabolic memory” or “Senescent memory” in cell senescence [41]. They found that metformin or resveratrol prevented senescent-memory by regulating the SIRT1/p300/p53/p21 pathway [41]. Treatment with resveratrol or metformin enhanced SIRT1 mediated signaling and was protective against senescent-memory, resveratrol or metformin are therefore promising anti-senescence drugs [41]. Studies by Huang et al. revealed aging as a dominant risk factor for worsening outcomes in acute MI patients [42]. Resveratrol is a calorie restriction mimetic [42]. In humans, nicotinamide phosphoribosyltransferase (NAMPT), telomerase reverse transcriptase (hTERT), sirtuin-4 (SIRT4) are activated by resveratrol in aortic smooth muscle cells (ASMCs), similar processes were observed in C57BL/6J mouse heart and liver cells treated with resveratrol [42]. Telomerase activity in human pulmonary endothelial cells and A549 Cells is increased by resveratrol [42]. It was observed that blocking SIRT4 and NAMPT expression prevented the induction of hTERT in ASMCs, while NAMPT overexpression elevated the telomerase activity already induced by resveratrol in the A549 cells [42]. By these results, it was identified that a NAMPT-SIRT4-hTERT axis is a mechanism by which the anti-aging process cardiovascular disease might be affected by resveratrol [42]. A study by Marin et al. showed that oxidative stress is a major cause of endothelial dysfunction, which is itself a major cause of cardiovascular disorders [43]. Oxidative cell damage causes telomere shortening, and telomere length corresponds to the consumption of a healthy diet [43]. Therefore cardiovascular diseases associated with aging may be a result of long-term oxidative stress influenced by dietary factors [43]. Studies by Csiszar et al. showed substantial evidence that age-related chronic low-grade inflammation increases the development of large-vessel diseases such as MI, stroke, peripheral arterial disease, and small vessel diseases like vascular dementia in the elderly [44]. Studies in Macaca mulatta primates showed the treatment of aged VSMCs (Vascular smooth muscle cells) with resveratrol (1 μM) reversed aging-associated changes in cellular cytokine production and inhibited NFk light chain enhancer of activated B lymphocytes, decrease in mitochondrial O(2)(-) production, increase in transcriptional activity of Nrf2 [44]. Resveratrol has been shown to prevent pro-inflammatory changes in aged VSMC secretome, this effect likely contributed to the vaso-protective effects of resveratrol in animal models [44]. The protective effects of resveratrol are shown in Figure 3.

Based on our data, we understand that resveratrol is a polyphenol found in red wine, derived mainly from grapes, and also a calorie restriction mimetic. The antioxidant, anti-inflammatory and cyto-protective effects of resveratrol have been specifically studied in cardiomyocytes. It has positive effects on mitochondrial health and integrity, and has been shown to induce degradation of impaired mitochondria in senescent cardiomyocytes. There is clear evidence that resveratrol slows cardiovascular aging. It is also protective against the formation of “Senescent memory” and has positive effects on slowing atherosclerosis and myocardial remodeling. It has been shown that resveratrol dose-dependently increases the activity of telomerase and increases endothelial progenitor cell (EPC) function, proliferation and inhibits EPC senescence.

## Conclusions

After reviewing 44 Articles specifically related to the context of the “effects of resveratrol on telomeres and post-myocardial infarction remodeling", we have concluded that resveratrol has been generally proven to be cardio-protective and vasculo-protective, it can be an effective therapeutic drug to counteract adverse myocardial remodeling following myocardial infarction or any other form of cardiac injury. Its effects on telomerase activation and preserving telomere integrity are essential in maintaining genomic stability in cardiac stem cells. Resveratrol's antioxidant, anti-inflammatory effects and its effects on mitochondrial degradation are essential in the maintenance of proper cardiomyocyte function as cardiomyocytes have a high concentration of mitochondria which are easily susceptible to oxidative injuries such as after ischemia or infarction. Resveratrol is a natural compound, hence can be extracted and supplemented easier than many synthetic compounds. It has the potential to become a mainstream therapeutic intervention for the prevention of a myriad of cardiovascular diseases from hypertension to post-MI remodeling and heart failure. We suggest that further research should be conducted to increase our current understanding of resveratrol, especially large-scale clinical trials in light of the immense scope it provides for therapeutic usage. We believe the answer to our “Question: Could resveratrol be to Myocardial infarction as Metformin is to diabetes mellitus?" is a resounding yes! 
